# Supplementary material for: Molecular surveillance reveals widespread colonisation by carbapenemase and extended spectrum beta-lactamase producing organisms in neonatal units in Kenya and Nigeria
Source: Antimicrob Resist Infect Control. 2023 Feb 22;12:14. doi: 10.1186/s13756-023-01216-0 (PMC9945588; doi:10.1186/s13756-023-01216-0)
Supplement: Supplementary file 1 — Additional file 1: Table S1. Occurrence of selected variables in participants according to colonisation with ESBL and CPOs. [file 13756_2023_1216_MOESM1_ESM.docx]

**Supplementary table 1.** Occurrence of selected variables in participants according to colonisation with ESBL and CPOs

| **Variable** | **ESBL [n (%)]** | | | | **CPO [n (%)]** | |  |
| --- | --- | --- | --- | --- | --- | --- | --- |
|  | **Colonised (n=36)** | **Uncolonised (n=6)** |  | **Colonised (n=28)** | | **Uncolonised (n=14)** | |
| Female | 18 (50.0) | 5 (83.3) |  | 17 (60.7) | | 7 (50.0) | |
| <28 weeks’ gestation | 5 (13.9) | 0 (0.0) |  | 2 (7.1.) | | 3 (21.4) | |
| Mother HIV positive | 2 (5.6) | 1 (16.7) |  | 2 (7.1) | | 1 (7.1) | |
| C section | 13 (36.1) | 5 (83.3) |  | 15 (53.6) | | 4 (28.6) | |
| Suspected sepsis | 25 (69.4) | 5 (83.3) |  | 17 (60.7) | | 13 (92.9) | |
| NEC | 4 (11.1) | 2 (33.3) |  | 5 (17.9) | | 1 (7.1) | |
| Mortality in NNU | 9 (25.0) | 3 (50.0) |  | 6 (21.4) | | 6 (42.9) | |

Notes: ^1^NEC; necrotising enterocolitis. ^2^NNU; neonatal unit.

ESBL = extended spectrum beta-lactamase

CPO = carbapenemase producing organism
